# The large-scale distribution of ammonia oxidizers in paddy soils is driven by soil pH, geographic distance, and climatic factors

**DOI:** 10.3389/fmicb.2015.00938

**Published:** 2015-09-04

**Authors:** Hang-Wei Hu, Li-Mei Zhang, Chao-Lei Yuan, Yong Zheng, Jun-Tao Wang, Deli Chen, Ji-Zheng He

**Affiliations:** ^1^State Key Laboratory of Urban and Regional Ecology, Research Center for Eco-Environmental Sciences, Chinese Academy of SciencesBeijing, China; ^2^Faculty of Veterinary and Agricultural Sciences, The University of MelbourneMelbourne, VIC, Australia

**Keywords:** barcoded pyrosequencing, ammonia oxidizers, spatial distribution, paddy soils, *Thaumarchaeota*, microbial biogeography, niche separation

## Abstract

Paddy soils distribute widely from temperate to tropical regions, and are characterized by intensive nitrogen fertilization practices in China. Mounting evidence has confirmed the functional importance of ammonia-oxidizing archaea (AOA) and bacteria (AOB) in soil nitrification, but little is known about their biogeographic distribution patterns in paddy ecosystems. Here, we used barcoded pyrosequencing to characterize the effects of climatic, geochemical and spatial factors on the distribution of ammonia oxidizers from 11 representative rice-growing regions (75–1945 km apart) of China. Potential nitrification rates varied greatly by more than three orders of magnitude, and were significantly correlated with the abundances of AOA and AOB. The community composition of ammonia oxidizer was affected by multiple factors, but changes in relative abundances of the major lineages could be best predicted by soil pH. The alpha diversity of AOA and AOB displayed contrasting trends over the gradients of latitude and atmospheric temperature, indicating a possible niche separation between AOA and AOB along the latitude. The Bray–Curtis dissimilarities in ammonia-oxidizing community structure significantly increased with increasing geographical distance, indicating that more geographically distant paddy fields tend to harbor more dissimilar ammonia oxidizers. Variation partitioning analysis revealed that spatial, geochemical and climatic factors could jointly explain majority of the data variation, and were important drivers defining the ecological niches of AOA and AOB. Our findings suggest that both AOA and AOB are of functional importance in paddy soil nitrification, and ammonia oxidizers in paddy ecosystems exhibit large-scale biogeographic patterns shaped by soil pH, geographic distance, and climatic factors.

## Introduction

Paddy ecosystems are essential components of the global agricultural systems, accounting for 75% of the worldwide rice production (Nicolaisen et al., 2004), and provide food to more than 50% of the world's population (Wang et al., 2014). Approximately 90% of the world's rice is cultivated in Asia, with China as the largest rice producing country (Lüke et al., 2014). As an anthropogenic aquatic ecosystem, paddy fields are characterized by intensive rice cropping practices (Hu et al., 2012), which received high levels of nitrogen-based fertilizers over the last decades, resulting in significant disturbance of the nitrogen-cycling processes (Bowatte et al., 2006). Autotrophic nitrification is a pivotal process of the global nitrogen cycle (Galloway et al., 2008), exerting significant control over the balance between relatively immobile ammonium and more mobile nitrite and nitrate, and thus is crucial for plant nitrogen availability and rice productivity. Simultaneously, nitrification can lead to significant losses of nitrogen fertilizers through groundwater nitrate leaching and greenhouse gas N_2_O production (Singh et al., 2010; Hu et al., 2015a), threatening the long-term sustainability of ecosystems security and services. There have been a large body of studies reporting the widespread occurrence of nitrification in the surface layers of water-logged paddy soils (Nicolaisen et al., 2004; Wu et al., 2011), however, considerable uncertainty remains about how environmental factors will affect nitrification processes in paddy soils.

Despite the fundamental importance of soil nitrification, few studies have addressed the phylogenetic composition and abundance of the key nitrifying microbes in paddy ecosystems (Ke et al., 2013; Wang et al., 2014). Among all the nitrogen cycling processes, ammonia oxidation is considered to be the first and the rate-limiting step for nitrification. This step is known to be performed by two distinct types of microbes: ammonia-oxidizing bacteria (AOB), belonging to two monophyletic groups within β- or γ-proteobacteria (Purkhold et al., 2000), and ammonia-oxidizing archaea (AOA), affiliated within the newly described *Thaumarchaeota* phylum (Brochier-Armanet et al., 2008). To date, the structure, abundance and diversity of AOA and AOB have been extensively examined in a range of upland soil environments including forest soils (Stempfhuber et al., 2014), drylands (Hu et al., 2013a), grasslands (Yao et al., 2013), and agricultural soils (He et al., 2007; Shen et al., 2008; Gubry-Rangin et al., 2011; Hu et al., 2014a). However, the dynamics and functioning of ammonia oxidizers in water-logged paddy ecosystems remain largely unexplored (Chen et al., 2008; Pett-Ridge et al., 2013). Compared with the well-documented upland soil ecosystems, paddy soils could be recognized a unique habitat for ammonia oxidizer adaptation to oxygen regimes, principally due to the low oxygen availability and fluctuating redox conditions caused by the flooding management during rice growth, which has a substantial impact on the structure and growth of indigenous ammonia oxidizers (Ke et al., 2013; Pett-Ridge et al., 2013). Therefore, it is essential to improve our understanding of the microbial ecology of ammonia oxidizers and their roles in paddy soils, which will eventually modulate the fate of nitrogen resources for plant.

Over the last several decades, there have been an ever-growing number of multi-scale studies investigating the spatial-temporal patterns of soil microbes and the underlying mechanisms (Griffiths et al., 2011; Shade et al., 2013). Aerobic ammonia oxidizers were considered to be an excellent model organism for studying microbial biogeography, due to their functional, numerical, and ecological importance, and the relative ease of characterization (Yao et al., 2013). A range of biotic and abiotic factors were distinguished to influence the ecological niches of ammonia oxidizers in upland soils, such as soil pH (Gubry-Rangin et al., 2011; Hu et al., 2013a; Oton et al., 2015), soil type (Chen et al., 2010), moisture contents (Hu et al., 2015b), temperature (Tourna et al., 2008), C/N ratios (Bates et al., 2011), sulfide (Erguder et al., 2009), and geographical distance (Hu et al., 2014a), however, relatively less effort was devoted to decipher the large-scale distribution patterns of ammonia oxidizers in paddy ecosystems. Simultaneously, numerous studies have demonstrated the cellular, genomic, and physiological differences between AOA and AOB (He et al., 2012; Prosser and Nicol, 2012), and their divergent nitrification pathways and responses to environmental and climatic factors (Tourna et al., 2008; Yao et al., 2013), which might lead to differential biogeographic patterns between AOA and AOB in paddy soils. Identifying factors driving the abundance and community composition of ammonia oxidizers could provide fundamental knowledge on the maintenance of ecosystem services in paddy fields, and the prediction of their responses to environmental disturbance.

The ongoing revolution of massively parallel sequencing technologies dramatically facilitate our understanding of the microbial dark matter, which has opened up the possibility of conducting broad-scale studies to survey the complex microbial communities spanning numerous samples (Hamady et al., 2008). The main objective of this study, therefore, was to examine the abundance, diversity, function, and community composition of AOA and AOB in 33 paddy soils collected from 11 major rice-growing regions along a latitudinal gradient in China, and to provide comprehensive insights into a range of geochemical, climatic, and spatial factors primarily driving the biogeographical patterns of the two microbial communities. We tested the following hypotheses: (1) in line with the extensive findings in upland soils, soil pH might be also the best predictor for the community compositions of AOA and AOB in paddy soils; (2) the community diversity of AOA and AOB might exhibit contrasting trends over the latitudinal gradient, owing to the general negative relationship between latitude and atmospheric temperature and the differential responses of AOA and AOB to temperature (Tourna et al., 2008); and (3) the biogeographic patterns of AOA and AOB would be regulated by both local (contemporary environment) and regional (historical contingencies) factors, because both of these two factors have shown significant impacts on microbial biogeography at the sampling scale similar to this study (Yannarell and Triplett, 2005; Ge et al., 2008).

## Materials and methods

### Soil sample collection

**Figure 1:**
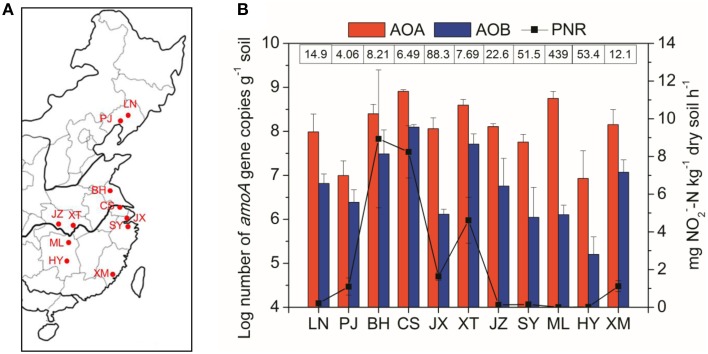
**(A)** Shows the paddy soil sampling sites in a regional map of China. Abbreviations in the map: LN, Liaoning; PJ, Panjin; BH, Binhai; CS, Changshu; JX, Jiaxing; XT, Xiantao; JZ, Jingzhou; SY, Shangyu; ML, Miluo; HY, Hengyang; XM, Xiamen. **(B)** Shows potential nitrification rates and abundance of AOA and AOB across the different paddy soil sites from North to South China. The numbers above the bars indicate the ratio of AOA to AOB *amoA* gene copies. Error bars represent standard errors (*n* = 3).

A total of 33 paddy soil samples were collected from 11 representative rice-cultivation regions across South and North China (24°61′N–41°52′N and 112°89′E–123°40′E) during May–August 2010 (Figure 1A). The sampling sites along a latitudinal gradient were chosen to span a wide spectrum of climatic and geochemical conditions, and the geographical coordinates were recorded using the Global Positioning System (GPS). All samples were collected during the rice-growing season when paddy fields were under flooded conditions. At each sampling site, three bulk soil samples (5 cm diameter) from the upper 20 cm between the rice plants were collected at a distance of 20 m from each other, and each sample was thoroughly homogenized by mixing five subsamples taken within an area of 50 m^2^. Soil samples were immediately transported on ice to the laboratory after collection, and separated into two portions upon arrival. The first portion was freeze-dried, passed through a 2.0-mm mesh, stored at −80°C prior to DNA extraction, and the second portion was stored at 4°C before determination of soil physicochemical properties.

### Physicochemical analysis and geographical distance

Soil pH was measured with a soil to water ratio of 2.5 using a Delta 320 pH-meter (Mettler-Toledo Instruments Co., Shanghai, China). Soil moisture was measured gravimetrically by oven-drying the samples at 105°C for 24 h. Total nitrogen (TN) was determined following the Dumas methods by an Element Analyzer (Vario EL III, Elementar, Hanau, Germany; Dumas, 1831). Inorganic nitrogen (ammonium and nitrate) was extracted from fresh soils with 1 M KCl immediately after sampling and measured colorimetrically by a Continuous Flow Analyzer (SAN++, Skalar, Breda, Holand; Maynard et al., 2007). Soil organic matter (OM) was determined using the K_2_Cr_2_O_7_ oxidation-reduction titration method (Walkley, 1947). Soil particle size was measured by the rapid sieving procedure (Kettler et al., 2001). Concentrations of sulfate (SO^2−^_4_) and chloride (Cl^−^) ions were analyzed using conventional methods on an Ion Chromatography (ICS2500, Dionex, USA; Binghui et al., 2006). The reasons for choosing these two ions were that sulfide produced from sulfate reduction and chloride ions have been reported to significantly impact the distribution of ammonia oxidizers (Chen et al., 2003; Erguder et al., 2009). The pairwise geographic distance was calculated by importing the GPS coordinates recorded at each sampling site into the NOAA website (http://www.nhc.noaa.gov/gccalc.shtml). The detailed information about the sampling locations, climatic conditions, geochemical properties, and pairwise geographic distances is listed in Tables S1 and S2.

### Potential nitrification rates (PNR) measurement

PNR was assessed immediately after soil sampling using the chlorate inhibition soil-slurry method (Kurola et al., 2005) with modifications, which represents the soil ammonia oxidation activity incubated with adequate ammonia substrate and oxygen within 24 h. Adjustment of soil slurry pH to 7.1 was thought to impair activity of indigenous acidophilic ammonia oxidizing strains (Prosser and Nicol, 2012), hence in this study, PNR was assessed at natural soil pH without adjustment of slurry pH. Briefly, for each sample, three subsamples (5 g of fresh soil) were incubated in 50 ml falcon tubes containing 20 ml of 1 mM (NH_4_)_2_SO_4_. Potassium chlorate was added to the tubes at a final concentration of 10 mg ml^−1^ to inhibit nitrite oxidation. The suspension was incubated at 25°C for 24 h in the dark, and nitrite was extracted with 5 ml of 2 M KCl and determined using a spectrophotometer at a wavelength of 540 nm with N-(1-naphthyl) ethylenediamine dihydrochloride (Hu et al., 2015b). PNR was calculated as the linear accumulation in nitrite concentrations during the incubation.

### DNA extraction and quantitative PCR (qPCR) analyses

Total genomic DNA was isolated from 0.5 g of frozen-dry soil using the Fast DNA® SPIN Kit for soil (MP Biomedicals, Cleveland, OH, USA) as per the manufacturer's instructions. The quantity and quality of the extracted DNA were checked using a NanoDropND-2000c UV-Vis Spectrophotometer (NanoDrop Technologies, Wilmington, DE, USA). Abundance of the AOA and AOB *amoA* genes (encoding the ammonia monooxygenase) was determined on an iCycler iQ5 thermocycler (Bio-Rad Laboratories, Hercules, CA, USA) using the primer pairs CrenamoA-23f/CrenamoA-616r (Tourna et al., 2008) and amoA-1F/amoA-2R (Rotthauwe et al., 1997), respectively. All qPCR reactions were conducted in triplicate with the extracted DNA from each sample. Each reaction was performed in a 25 μl volume containing 2 μl of five-fold diluted template DNA (1–10 ng), 0.25 μl of each primer (10 μM), and 12.5 μl of SYBR® Premix Ex Taq™ (TaKaRa Biotechnology, Dalian, China). Thermo-cycling conditions were as follows: 2 min initial denaturation step at 95°C, 35 cycles of 10 s at 95°C, 30 s at 53°C for AOA and 55°C for AOB, and 1 min at 72°C, followed by a plate read at 83°C. A 10-fold dilution series of standard curves for the qPCR assays were generated using putative AOA and AOB clones as described previously (He et al., 2007). Melting curve analysis was performed at the end of each qPCR run to check the specificity of amplicon products, before confirmation by standard agarose gel electrophoresis. Amplification efficiency ranged between 89 and 99%, with *R*^2^-values of 0.997–0.999 for all assays.

### High-throughput 454-FLX pyrosequencing

Pyrosequencing of the AOA and AOB *amoA* genes were performed with the primers Arch-amoF2/Arch-amoAR (Nelson et al., 2010) and amoA-1F/amoA-2R, respectively, as described by Hu et al. (2013a). The primer sets were modified by adding the Roche 454 adaptor A followed by 10-nucleotide unique barcode sequences to the 5′-end of the forward primers, and adding the adaptor B to the 3′-end of the reverse primers. The thermal-cycling conditions were used as follows: 2 min initial denaturation at 95°C, 35 cycles of 30 s at 95°C, 1 min at 55°C for AOA, and 58°C for AOB and 1 min at 72°C, ended with an elongation of 7 min at 72°C. Triplicate independent PCR reactions (50 μl each) were performed for each sample and the resulting PCR products were mixed together, and purified using a Wizard SV Gel and PCR Clean Up Kit (Promega, San Luis Obispo, CA, USA). The concentrations of PCR products were quantified using a Quant-iT dsDNA HS Assay Kit (Invitrogen, Carlsbad, CA, USA). The purified PCR products were combined in approximately equimolar amount into a single tube, and send for 454 amplicon sequencing from the fusion adapter A on a Roche 454 GS FLX Titanium platform (Roche Diagnostics, Branford, CT, USA).

### Processing of pyrosequencing data

Bioinformatic analyses were conducted as described previously (Hu et al., 2013a) using the Mothur platform, version 1.34.1 (Schloss et al., 2009). Briefly, raw pyrosequencing reads shorted than 400 bp in length, with ambiguous nucleotides and average quality scores lower than 30 were eliminated to improve sequence quality. All reads with the same barcode were assigned to each sample, and then the barcode and primer sequences were removed from the dataset. The *amoA* gene sequences were aligned against the ARB databases for AOA and AOB created by Abell et al. (2012) via the *align.seqs* command within Mothur, and further quality trimmed to remove putative chimeric sequences by performing the *chimera.uchime* algorithm (Edgar et al., 2011). The sequencing noise due to sequencing errors was further reduced by using the *pre.cluster* command within Mothur. After these quality-control processes, high-quality sequences including a total of 171,891 reads for AOA, and 59,629 reads for AOB were used for downstream analysis.

To correct for differences in the sampling efforts, a randomly selected subset of 1000 and 347 sequences per sample for AOA and AOB, respectively, was used to calculate alpha diversity and the community dissimilarity between samples. The resampling strategy was selected to retain the maximal number of soil samples with the maximal number of reads, which resulted in 27 and 25 samples for AOA and AOB, respectively. Alpha diversity of AOA and AOB was estimated by calculating the operational taxonomic unit (OTU) richness and Shannon diversity indices at the 85% sequence identity (Purkhold et al., 2000; Pester et al., 2012). A set of representative sequences was retrieved from each OTUs for the taxonomic assignment of the major lineages of AOA and AOB. Phylogenetic identities of the representative sequences were determined by creating neighbor-joining trees using Kimura 2-parameter distance including the taxonomy-determined reference sequences from the National Centre for Biotechnology Information (NCBI) database, within MEGA version 6.0 (Tamuka et al., 2013). Bootstrap analysis with 1000 replicates were performed to estimate the confidence values for the tree nodes. The sequences with ambiguous taxonomy were excluded along with their OTUs, and and the taxonomy was assigned to each OTU with robust phylogenetic supports. The neighbor-joining phylogenetic trees for AOA and AOB *amoA* genes are shown in the supplementary materials (Figures S1, S2). The pyrosequencing reads of the AOA and AOB *amoA* genes have been deposited in the DNA Data Bank of Japan under the accession number DRA003908.

### Statistical analysis

Spearman's correlation analyses were performed to assess the relationships between geochemical properties, PNR, the log-transformed AOA and AOB abundances, and the relative abundance of specific ammonia-oxidizing lineages. The relationships between the taxonomic diversity and geochemical properties were tested with linear regression analysis within the SPSS version 19.0 (IBM Co. Armonk, New York, USA). The Bray–Curtis dissimilarity matrices of AOA and AOB were calculated based on the OTUs at the 85% identity level, and correlated with the pairwise geographical distances using the linear regression. *P* < 0.05 was considered to be significantly different. Geochemical data were standardized to make the different factors comparable (Xiong et al., 2012). Canonical correspondence analysis (CCA) was performed to identify the major climatic and geochemical factors driving the differences in overall community compositions of ammonia oxidizers with latitude, MAT, MAP, soil pH, H_2_O%, OM, TN, C/N, NH^+^_4_–N, NO^−^_3_–N, sand%, clay%, sulfate, and chloride as the explanatory factors. Only the factors which had significant effects on the community compositions were retained for the biplots of the CCA analysis.

A redundancy analysis (RDA)-based variation partitioning analysis was conducted to evaluate the relative importance of geochemical, climatic and spatial characteristics on the community compositions of both AOA and AOB (Peres-Neto et al., 2006; Tripathi et al., 2015). The Hellinger-transformed OTU abundance data were used as response variables for this analysis. Geochemical variables included soil pH, moisture content, TN, OM, C/N ratio, sulfate, chloride, ammonium, nitrate, clay%, and sand%, while climatic variables included MAT and MAP. The principal coordinates of neighbor matrices (PCNM) were calculated to model the spatial structure between the 33 paddy soil samples (Dray et al., 2006). The importance of climatic, geochemical, and spatial factors in explaining the community compositions was determined by a RDA analysis using Monte Carlo permutation test with 999 unrestricted permutations, followed by forward selection to include only the PCNM variables that explain significant variation in the response data. All these variation partitioning analyses were performed as described previously (Tripathi et al., 2015) using *Vegan, ape, packfor, AEM, PCNM*, and *ade4* packages in R platform (http://www.r-project.org).

## Results

### Soil geochemical properties, climatic characteristics, and PNR

A wide variety of geochemical and climatic parameters of the paddy soil samples are summarized in Table S1. These samples represented a large spatial scale with the pairwise geographical distance between sampling sites ranging from 75 to 1975 km (Table S2). Soil pH highly varied from 5.04 in ML to 8.77 in PJ across the 33 paddy soils, with the water contents ranging between 33.7 and 86.2%. The soil samples also considerably differed with respect to OM from 9.6 to 42.0 g kg^−1^, TN from 0.41 to 2.72 g kg^−1^, NH^+^_4_–N from 6.93 to 84.6 mg kg^−1^, NH^−^_3_–N from 0.04 to 5.26 mg kg^−1^, and sulfate from 23.7 to 355 mg kg^−1^. There was also a large variation in climatic conditions which ranged from 8.1 to 21.0°C for MAT, and from 650 to 1400 mm for MAP (Table S1). Spearman's correlation analysis found that latitude had significant relationships with climatic factors (MAT and MAP) and most of the measured soil properties (Table S3). There were also significant relationships between climatic factors and soil properties, and within soil properties (Table S3). The great variability in geochemical properties and climatic conditions is in coincidence with changes in PNR from 0.01 to 13.0 mg NO^−^_2_–N kg^−1^ dry soil h^−1^ (Figure 1B), with the highest value recorded in BH (pH 8.18). Spearman's correlation analysis revealed that PNR was significantly and positively correlated with soil pH, sulfate, chloride and latitude, and negatively correlated with climatic parameters including MAT and MAP (Table S4).

### Abundance of AOA and AOB in paddy soils

The AOA *amoA* gene abundance was in the range of 1.21 × 10^7^ to 8.09 × 10^8^ copies per g of dry soil, with the highest values recorded in the CS site (Figure 1B). The AOB *amoA* gene copies varied by more than three orders of magnitude from 2.03 × 10^5^ to 1.25 × 10^8^ copies per g of dry soil. The AOA abundance was consistently higher than AOB, resulting in the ratios of AOA to AOB *amoA* gene copies ranging from 4.06 in the PJ site to 439 in the ML site. The abundances of both AOA and AOB were significantly and positively correlated with PNR (Table S4), indicating their potential important roles in paddy soil nitrification. In addition, AOA abundance was significantly affected by TN and sand%, while AOB abundance was significantly correlated with MAP and soil pH (Table S4).

### Taxonomic classification of paddy soil AOA and AOB

**Figure 2:**
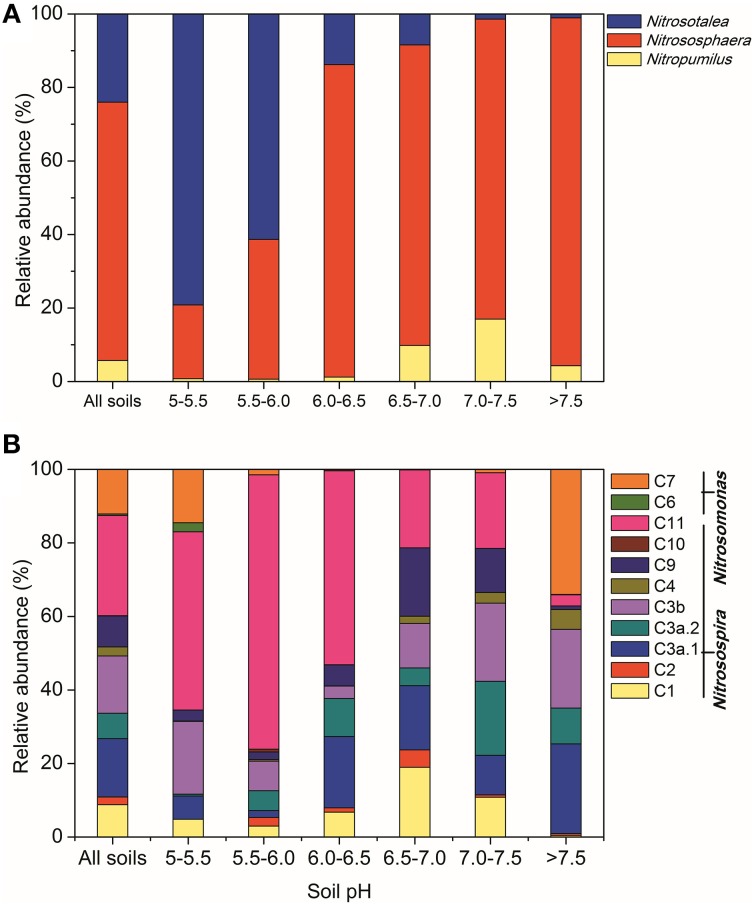
**Relative abundances of different lineages of AOA (A) and AOB (B) in all soils combined and in soils with different pH categories, based on the pyrosequencing data of the AOA and AOB *amoA* genes, respectively**.

Across all the paddy soil samples, barcoded pyrosequencing produced a total of 171,891 high-quality reads for the AOA *amoA* gene, with an average of 5209 sequences per sample. The most dominant AOA lineages was *Nitrososphaera* (accounting for 70.3% of the obtained AOA sequences), followed by *Nitrosotalea* (24.0%; Figure 2A). The remaining AOA sequences (5.7%) were classified into the *Nitrosopumilus* lineage, which was found to be present in low abundance in most of the examined paddy soils. The differentiations of the relative abundance of AOA lineages were strongly affected by a range of geochemical and climatic parameters, but could be best explained by soil pH (Table S4). For example, the *Nitrosotalea* lineage was much more abundant in paddy soils with lower pH-values (< 6.0), and tended to decrease with the increasing soil pH, while the *Nitrosopumilus* lineage showed an inverse trend and were relatively more prevailing in neutral and alkaline soils (Figure 2A). The *Nitrososphaera* lineage predominated in the AOA community in paddy soils with pH > 6.0, but was a minor group in acidic soils (Figure 2A). Interestingly, the relative abundance of *Nitrososphaera* was found to be significantly and positively correlated with PNR (Table S3), suggesting the potential functional importance of this abundant lineage in paddy soil nitrification.

A total of 59,629 high-quality reads were obtained for the AOB *amoA* gene, with an average of 1807 sequences for each sample. The majority of AOB sequences across all the paddy soils were classified into the *Nitrosospira* lineage (accounting for 87.5% of the obtained AOB sequences) including nine clusters (Figure 2B), of which Cluster 11 was the most abundant group (27.3%), followed by Clusters 3a.1 (15.9%), 3b (15.6%), 1 (8.8%), 9 (8.4%), 3a.2 (6.9%), 4 (2.4%), and 2 (2.1%). The *Nitrosomonas* lineage including Clusters 6 and 7 comprised 12.5% of the total AOB sequences, and was found to be favored in either strongly acidic soils (pH < 5.5) or alkaline soils (pH > 7.5; Figure 2B). Although multiple factors were found to significantly affect the differentiation of AOB clusters (Table S5), we could still observe a clear trend of the relative abundance of AOB clusters along the pH gradient (Figure 2B). Notably, Cluster 11 was much more abundant in acidic soils (pH < 6.5) occupying up to 74.7% of the AOB communities in soils with pH 5.5–6.0, while Clusters 1, 3a.1, 3a.2, 3b, and 9 tended to be more adapted to thrive in neutral and alkaline soils. In addition, the relative abundances of Clusters 3a.1, 3a.2, 3b, and 4 (belonging to *Nitrosospira*) had significantly positive correlations with PNR, indicative of their potential contribution to paddy soil nitrification (Table S5).

### Alpha diversity of AOA and AOB

**Figure 3:**
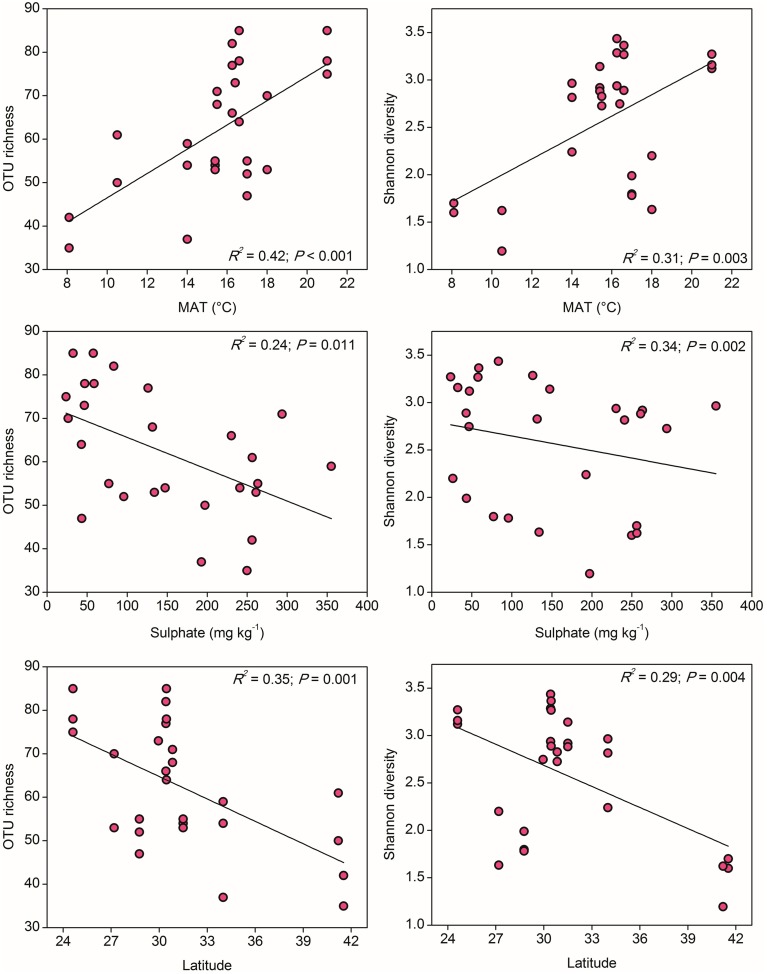
**The linear relationships between OTU richness or Shannon diversity of AOA and Mean Annual Temperature (MAT), sulfate, and latitude**. The AOA community was normalized at 1000 randomly selected *amoA* gene sequences for calculation of alpha diversity at 85% sequence similarity.

To compare the community diversity of ammonia oxidizers in paddy soils, the survey effort was normalized to 1000 and 347 randomly selected sequences for AOA and AOB for each sample, respectively. Alpha diversity of AOA, measured as OTU richness and Shannon diversity indices at the 85% similarity level, showed evident variations across the examined paddy soils. Spearman's correlation analysis demonstrated that, among all the climatic and geochemical characteristics, AOA alpha diversity was significantly correlated with MAT, sulfate, and latitude (Table S6). Clear trends for the alpha diversity indices of AOA could be observed along the gradients of these three parameters, if fitted with the linear regression relationships (Figure 3). The values of OTU richness and Shannon diversity for AOA significantly increased with the increasing MAT, and as expected, decreased with the increasing latitudinal gradients, while the diversity indices were negatively influenced by the concentrations of sulfate.

**Figure 4:**
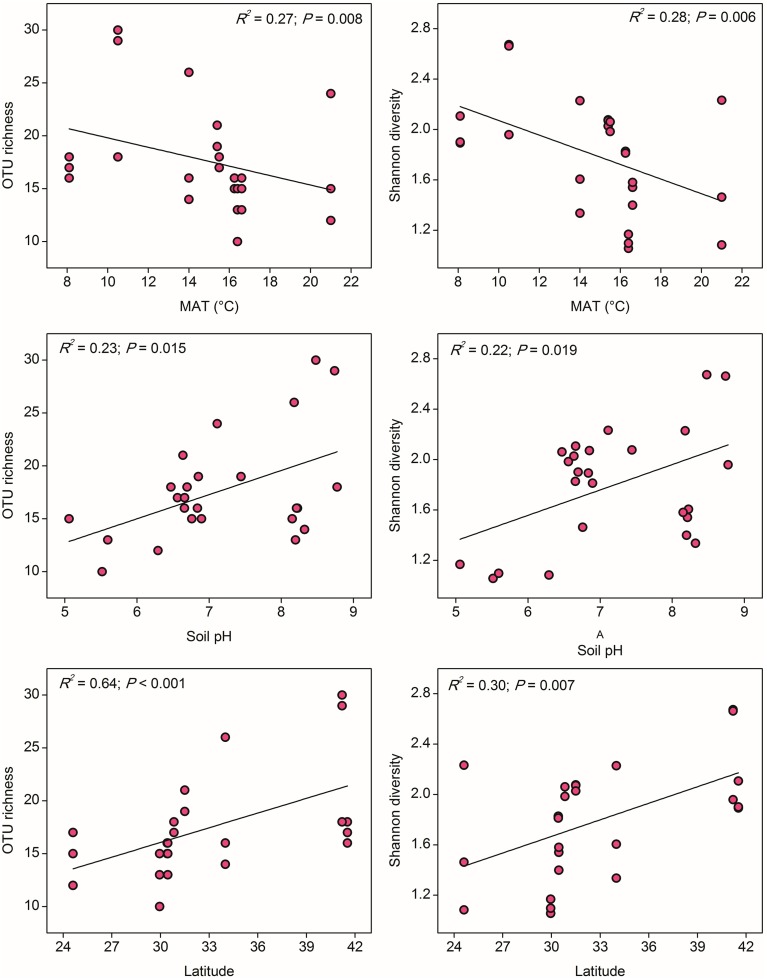
**The linear relationships between OTU richness or Shannon diversity of AOB and Mean Annual Temperature (MAT), soil pH, and latitude**. The AOB community was normalized at 347 randomly selected *amoA* gene sequences for calculation of alpha diversity at 85% sequence similarity.

In contrast to the results for AOA, the distribution of AOB alpha diversity at the 85% similarity level was positively related to latitude and negatively related to MAT, which suggested a possible niche separation between AOA and AOB shaped by the gradient of latitude due to changes in MAT (Table S6). AOB alpha diversity was significantly and positively correlated with soil pH (Figure 4), indicating that soil pH has more pronounced effect on the AOB community diversity compared with the AOA (no significant relationship was found between AOA diversity and soil pH). No significant correlations could be observed between AOB diversity with other climatic and geochemical characteristics (Table S6).

### Impacts of environmental factors on the community structure of AOA and AOB

**Figure 5:**
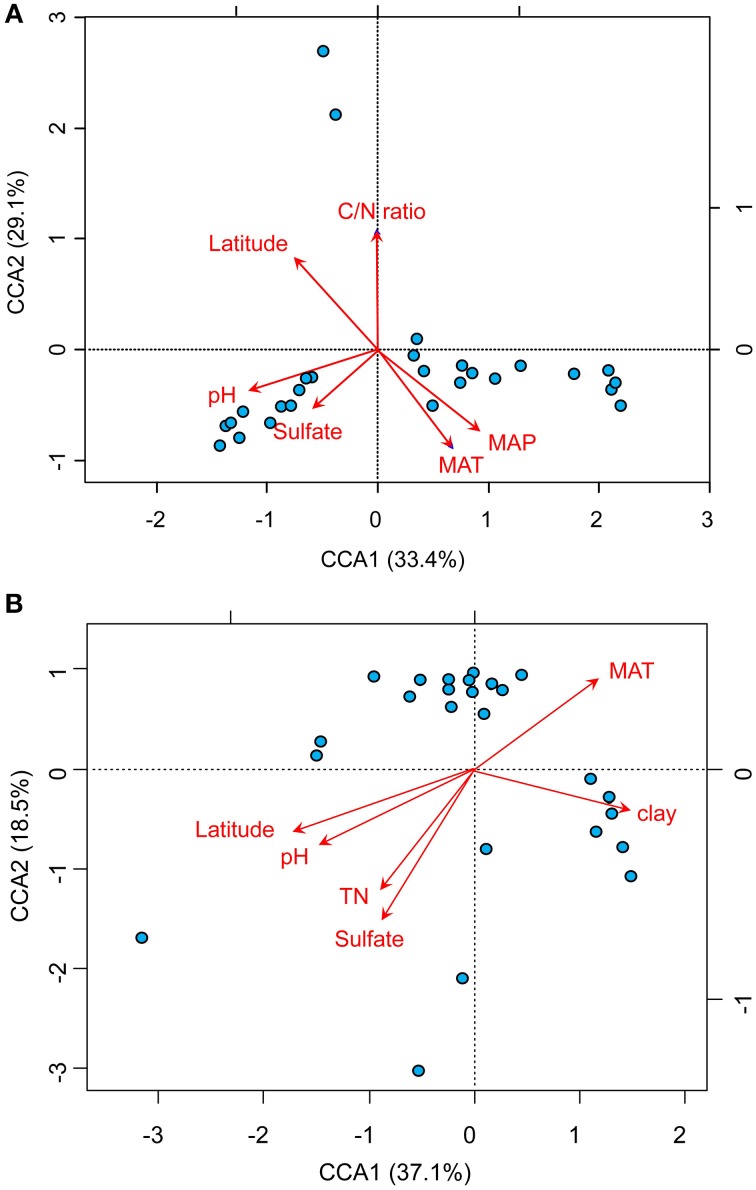
**Biplots of canonical correspondence analysis of environmental factors and pyrosequencing data of AOA (A) and AOB (B)**. A random subset of 1000 and 347 sequences per sample was used for AOA and AOB, respectively. The red arrows indicate the vectors of the explanatory variables which have significant effects on ordination of the communities of AOA and AOB. The blue spots indicate the sampling sites for paddy soils. (MAT, mean annual temperature; MAP, mean annual precipitation).

**Figure 6:**
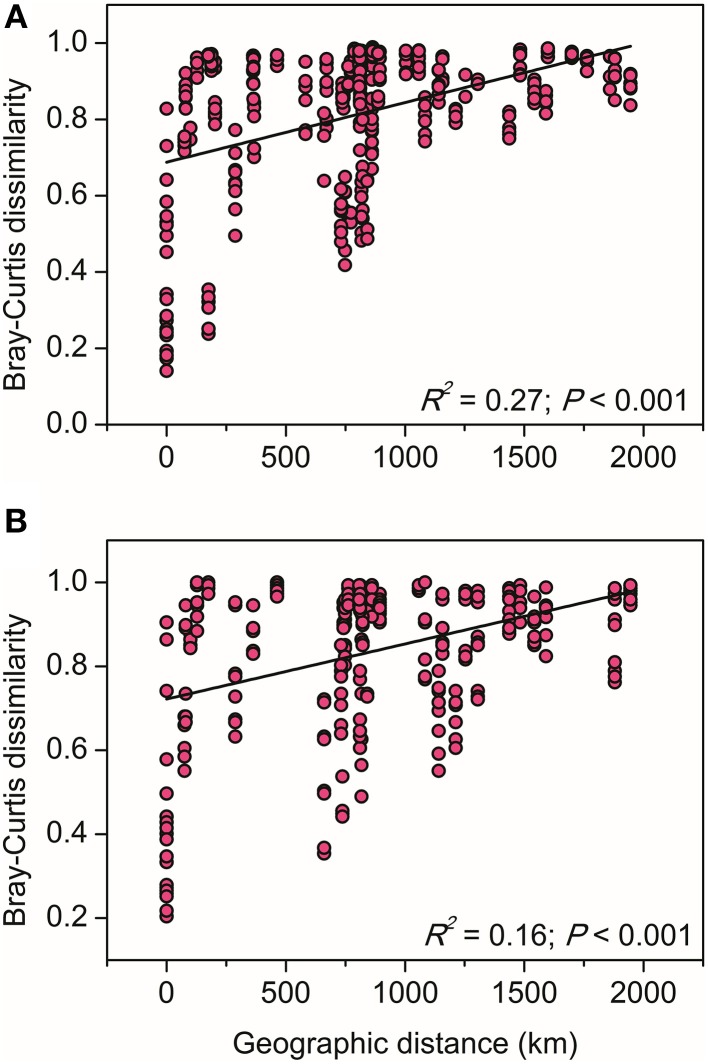
**The linear relationships between geographic distance and the Bray–Curtis dissimilarity of AOA (A) and AOB (B) communities**.

The relationships between the community structure of ammonia oxidizers with latitude, geochemical and climatic factors in paddy soils was assessed with a CCA analysis, which revealed clear differences in both AOA and AOB communities across the sampling sites (Figure 5). Of all the environmental factors, soil pH had the most significant effect on the biogeographic distribution of AOA in paddy soils, followed by latitude, MAT, and MAP (Figure 5A). Other factors including C/N ratios and sulfate also significantly contributed to separation of AOA communities in different sampling sites. With respect to AOB communities, latitude, soil pH, and sulfate were the most important determinants driving the distribution patterns (Figure 5B), which were also significantly influenced by MAT, clay%, and TN. Furthermore, distance-decay curves were constructed to explore the relationships between the Bray–Curtis dissimilarities of AOA and AOB and pairwise geographical distances. The results revealed that the community dissimilarity of both AOA and AOB was significantly and positively correlated with geographical distance (Figure 6), indicating that spatial isolation of the sampling sites might affect ammonia oxidizer community structure.

**Figure 7:**
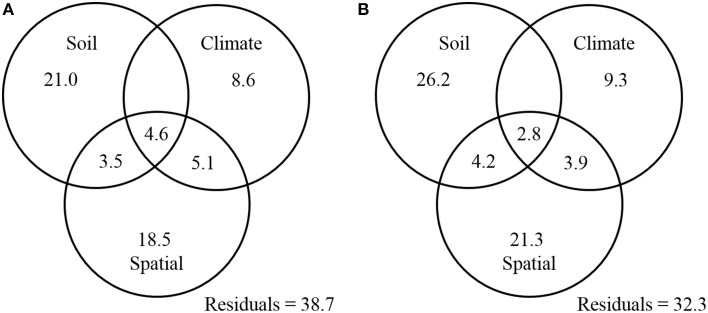
**Variation partition analysis of the effects of soil properties, climatic parameters, spatial factors, and their interactions on the community structure of AOA (A) and AOB (B)**. The data represent percentages of variation explained by the factors.

Finally, a variance partitioning analysis was conducted to assess the relative contributions of soil properties, climatic conditions, and spatial factors to the distribution patterns of ammonia oxidizers. The combination of these variables explained 61.3 and 67.7% of the observed community variation in AOA and AOB, respectively. With respect to AOA, soil properties (including soil pH, C/N ratio, and sulfate) significantly explained 29.1% of the total variation, of which 21.0% was not related to climate or spatial factors (Figure 7A). Climatic factors (including MAT and MAP) and spatial factors (as represented by the PCNM variables) could explain 18.3 and 31.7% of the total variation of AOA communities, respectively, with their unique percentages of explanations confined to 8.6 and 18.5%, respectively (Figure 7A). Likewise for the AOB community, soil variables (including soil pH, TN, and sulfate) explained the largest portion (33.2%) of the variation, followed by spatial factors (32.2%), and climatic factors (16.0%) (Figure 7B).

## Discussion

Although numerous studies have reported the biogeographic distribution of ammonia oxidizers in upland soils (Gubry-Rangin et al., 2011; Hu et al., 2013a; Yao et al., 2013), this study, to our knowledge, represents the first large-scale pyrosequencing investigation of AOA and AOB in water-logged paddy soil agroecosystem. In this study, we presented a large-scale investigation into the ammonia-oxidizing communities across 11 major rice-growing regions along a latitudinal gradient, and generated a panoramic view of these important guilds and their biogeographic patterns. We provide strong evidence that ammonia oxidizers in paddy soils are not freely dispersed, and they do exhibit microbial biogeographical patterns. Correlative evidence revealed that the abundances of both AOA and AOB, and several major lineages of ammonia oxidizers were significantly and positively correlated with PNR, suggesting their potential functional importance in paddy soil nitrification. However, it should be noted that: (1) the paddy soil samples were collected within a time frame of 4 months, the possible differences in air temperatures and growth stages of plants across the sampling sites may be overlooked factors explaining the observed biogeographical patterns of ammonia oxidizers; and (2) the patterns of ammonia oxidizer diversity and distribution were based merely on the genetic potential of ammonia oxidation, which does not mean these populations are really functioning and demonstrate a similar pattern of ammonia oxidation activity in paddy soils.

### Soil pH as a prominent factor influencing community compositions of ammonia oxidizers in paddy fields

Despite the striking differences in physicochemical properties between upland and paddy soils (Hu et al., 2013b), the abundance and community compositions of ammonia oxidizers in the examined paddy soils are not highly divergent from previous studies in upland soils (Pester et al., 2012; Hu et al., 2013a). In accordance with previous findings in a wide variety of upland soil ecosystems (Gubry-Rangin et al., 2011; Shen et al., 2012; Zhang et al., 2012; Hu et al., 2013a), where different ammonia-oxidizing ecotypes have evolved to thrive at different pH categories, our study further expanded the pH-structured ecological niches of the major ammonia-oxidizing lineages into the water-logged paddy soils (Figure 2). The underlying mechanisms were generally attributed to the pH-impacted bioavailability of multiple elements, such as ammonia, aluminum, manganese, copper, iron, sulfur, and phosphorus, and the intrinsic biochemical features of ammonia oxidizers, which have been comprehensively discussed and reviewed previously (He et al., 2012; Hu et al., 2014b). The direct or indirect pH-associated influences might result in the observed changes of ammonia oxidizers and thus the pH-associated changes of PNR in paddy ecosystems.

A large body of pyrosequencing studies have shown that *Nitrososphaera* was the most dominant AOA lineage in upland soils and tended to thrive under neutral and alkaline conditions (Gubry-Rangin et al., 2011; Pester et al., 2012; Hu et al., 2013a). Likewise in paddy ecosystems, *Nitrososphaera* overwhelmingly dominated in neutral and alkaline soils, and seemed sensitive to low pH, but still comprising a considerable proportion in most of the acidic soils (Figure 2A). Genomic annotations of the *Nitrososphaera* strains also suggested their genomic capacity of using various ammonia sources and flexible carbon metabolism, facilitating their metabolic versatility and physiological adaptations under various environmental conditions (Spang et al., 2012). We further found that the relative abundance of *Nitrososphaera* was significantly and positively related to PNR (Table S3), suggesting their potential involvement in paddy soil nitrification. Therefore, we supposed that numerously abundant *Nitrososphaera* might be also functionally important, particularly in neutral and alkaline paddy soils. Another dominant AOA lineage *Nitrosotalea* was generally found to be functionally active in acidic soils (Zhang et al., 2012), and has been recognized as an obligate acidophilic lineage (Lehtovirta-Morley et al., 2011). Our study demonstrated similar features of *Nitrosotalea* in acidic paddy soils irrespective of the oxygen-limited conditions, further confirming their high-level tolerance of acidity. In addition, although *Nitrosopumilus* were marine-originated and expected to be abundant in water-logged paddy soils, we found that this lineage represented only a minor group, was also strongly impacted by soil pH, and tended to be more adapted to high-pH conditions.

The AOB community was mainly composed of the two genera *Nitrosomonas* and *Nitrosospira* belonging to β-proteobacteria across the examined paddy soils (Figure 2B). The numerical predominance of *Nitrosospira* over *Nitrosomonas* (Figure 2B) is consistent with findings in upland soils (Yao et al., 2013), and four clusters of the *Nitrosospira* lineage (including Clusters 3a.1, 3a.2, 3b, and 4) were significantly and positively correlated with PNR (Table S4), further indicating the functional potential of *Nitrosospira* in paddy ecosystems. Five clusters of AOB were significantly related to soil pH, wherein Cluster 11 was primarily obtained from acidic soils, while Clusters 3a.2, 3b, 4, and 7 were predominantly distributed in neutral and alkaline soils (Figure 2B). Intriguingly, Cluster 11, which was found to be a minor group in upland soils (Hu et al., 2013a), was the most dominant AOB cluster in paddy soils, comprising 27.3% of all the obtained AOB sequences, and has extraordinary capacity to thrive in acidic and neutral soils. The majority of the *Nitrosomonas* lineage was found in neural paddy soils in a previous study (Li et al., 2015). However, we found that *Nitrosomonas* (including Clusters 6 and 7) can be present in relatively higher abundance in either strongly acidic soils (pH < 5.5) or in alkaline soils (pH > 7.5) (Figure 2B). All these findings provided broad-scale evidence for the principal role of soil pH in structuring the ecological niches occupied by specific AOA and AOB lineages in paddy soils, which is tightly associated with the pH-impacted nitrification activity as measured by PNR.

### Community diversity of paddy soil ammonia oxidizers along the latitudinal gradient

It has been long documented that the diversity of macro organisms like plants and animals decreases from the equator to the poles along the increasing latitudinal gradient (Willig et al., 2003). Soil bacterial diversity was also occasionally reported to decrease with increasing latitude (Xiong et al., 2012) and correlated positively with atmospheric temperature (Staddon et al., 1998), while some other studies argued that soil pH, rather than latitude and annual temperature, was the best predictor for microbial diversity (Fierer and Jackson, 2006). In our study, the community diversity of both AOA and AOB was significantly correlated with latitude and MAT, but showed contrasting trends over the gradients (Figures 3, 4). In fact, the latitude itself was expected to have no effect on the diversity of ammonia oxidizers, however, higher ambient temperature at low latitudes (rather than evolutionary history or geographical constraints) was assumed to be a probable mechanism underlying and predicting the latitudinal pattern of richness (Fuhrman et al., 2008; Soininen, 2012). Given the significantly negative relationship between latitude and MAT in this study (Table S3), we suppose that the large-scale latitudinal pattern of the ammonia oxidizer diversity might be driven by the changes in atmospheric temperature. However, it should be noted that latitude was also significantly correlated with MAP and other soil properties such as soil pH, H_2_O%, OM, TN, sulfate, and chloride (Table S3), and the diversity of AOA and AOB was observed to be strongly related to sulfate and soil pH, respectively (Figures 3, 4). These factors might also contribute to the large-scale latitudinal biogeographic patterns of AOA and AOB in paddy soil ecosystems.

Although AOA and AOB diversity was significantly correlated with MAT, they showed distinct trends along the latitudinal gradient, for instance, AOA tended to be more diverse in regions with higher MAT, while AOB were in reverse. The contrasting latitudinal patterns of AOA and AOB diversity might be driven by their intrinsic physiological adaptation to temperature. Substantial evidence has suggested that isolated AOA strains generally have higher temperature optima than their counterparts AOB. For instance, growth of the soil AOA strain *Nitrososphaera viennensis* is favored at 37°C (Tourna et al., 2011), the moderate thermophilic Candidatus *Nitrososphaera gargensis* shows optimal growth under 46°C (Hatzenpichler et al., 2008), and the thermophilic AOA *Nitrosocaldus yellowstonii* could grow up to 74°C (de la Torre et al., 2008). A maximum growth rate of the marine AOA strain *Nitrosopumilus maritimus* was observed at 32°C, but no growth could be detected at 10°C (Qin et al., 2014). By contrast, to date no AOB strains have been isolated from environments with constant temperatures above 40°C (Jiang and Bakken, 1999; Hatzenpichler, 2012), and AOB were found to be favored under ambient temperature, compared to the adaptation of AOA to elevated temperature (Tourna et al., 2008; Hu et al., unpublished data). Therefore, their differential responses to temperature might drive a large-scale niche separation of AOA and AOB along the latitudinal gradient, and AOA might have an ecological advantage over their counterparts AOB in paddy soils with higher MAT.

### Biogeographic patterns of AOA and AOB in paddy soils are regulated by historical contingencies and contemporary environment

The decrease of community similarity with increasing geographic distance (the distance-decay patterns) is regarded as a general phenomenon in macro organisms (Nekola and White, 1999), which has been also discovered for bacteria in a variety of terrestrial, lake and marine habitats (e.g., Martiny et al., 2011; McAllister et al., 2011; Xiong et al., 2012). In contrast to these studies in natural settings, we focused here on functional communities within the rice agroecosystem managed by intensive agricultural practices and dominated by one plant species during the rice-growing season. We demonstrated that ammonia oxidizers in large-scale paddy fields exhibit the classical distance-decay patterns as those observed for methanotrophic communities in wetland rice fields (Lüke et al., 2014). Such patterns could be potentially explained by two ecological processes: environmental selection (different environmental conditions across space) and dispersal limitation, or a combination of both processes (Soininen, 2012). Firstly, different from the marine environments with more homogeneous structure enabling virtually free dispersal of microorganisms (Soininen, 2012), microorganisms in paddy soils appear more fragmented and are expected to be limited in dispersal (Martiny et al., 2006). In our study, not all phylotypes of AOA and AOB could be found in all sampling sites (Figure 2), suggesting that dispersal limitation indeed exist in paddy soil at large scales. Although AOA are locally more abundant and differ significantly in cellular sizes and physiological features from AOB (He et al., 2012; Hu et al., 2014b and references therein), we observed comparable distance-decay relationships for them. Secondly, the paddy fields examined in this study are located at large spatial scales across different climatic regions and encompassing a broad gradient of geochemical factors (Table S1). The impacts of these climatic and geochemical factors in distribution of ammonia oxidizers were indicated by the CCA and variation partitioning analysis, which led us to presume a potential effect of environmental selection. However, the relative contributions of these two ecological processes remain unknown and desire further examination.

Recent studies identified soil pH (Gubry-Rangin et al., 2011; Hu et al., 2013a; Tripathi et al., 2015) and spatial locations (Hu et al., 2014a) as the two most important drivers of biogeography of ammonia oxidizers in upland soils. Other studies suggested that no single factor could fully explain the function and adaption of ammonia oxidizing populations in complex natural settings (Yao et al., 2013). The major influential factors driving the present prokaryotic biogeographical patterns could be generally classified into contemporary environmental conditions and historical contingencies (Martiny et al., 2006; Ge et al., 2008). The relative importance of historical (spatial) factors and contemporary environmental (geochemical and climatic) factors on the distribution of microbial assemblages has been explored in upland soils (Ge et al., 2008) and aquatic environments (Vyverman et al., 2007). In this study, CCA analyses suggested that the biogeographic patterns of both AOA and AOB were significantly regulated by multiple factors including spatial factors (latitude), climatic factors (MAT and MAP), and geochemical factors (soil pH, TN, sulfate, clay%, and C/N ratio) (Figure 5). Variation partitioning analysis further suggested that spatial, geochemical, and climatic factors could collectively explain 61.3 and 67.7% of the variation in AOA and AOB communities, respectively (Figure 7). In fact, historical contingencies, referring to past evolutionary and ecological events such as physical barrier and dispersal history, would not directly influence ammonia oxidizers, but was related to the probability of past divergence and diversification of microbial assemblages (Ge et al., 2008). The relative influence of historical contingencies and contemporary environment appears to be related to the sampling scale (Martiny et al., 2006). At intermediate scales (10–3000 km), some studies found that both of these two factors could influence the community compositions of bacteria (Yannarell and Triplett, 2005; Ge et al., 2008). The sampling scale (75–1945 km) of this study was well-fell within the intermediate scales, therefore, it is not surprising to observe that ammonia oxidizers exhibit biogeographical patterns regulated by both local (contemporary environment) and regional (historical contingencies) factors.

## Conclusions

In conclusion, by pyrosequencing the ammonia-oxidizing microorganisms in water-logged paddy soils collected from 11 major rice-growing regions in China, this study comprehensively demonstrated that paddy soil ammonia oxidizers are not randomly distributed over space, they do exhibit large-scale biogeographic patterns similar to those of the well-documented macro organisms. Soil pH is the predominant factor shaping the distribution of the major lineages of ammonia oxidizers in paddy fields, echoing the findings from a wide variety of upland soil ecosystems. The classical distance-decay relationships exist for ammonia oxidizers, and we could clearly see that ammonia-oxidizing communities in paddy fields become more dissimilar with increasing geographical distance. The biogeographic patterns of paddy soil ammonia oxidizers could be jointly explained by spatial, climatic and geochemical factors, and thus highlighting the importance of multiple factors on shaping the observed patterns. Finally, we found that PNR was significantly and positively correlated with the abundances of both AOA and AOB, and with several major ammonia oxidizer lineages (including the *Nitrososphaera* lineage of AOA, and the Clusters 3a.1, 3a.2, 3b, and 4 of AOB), suggesting their potential functional importance in paddy soil nitrification. Future research is desirable to develop a theoretical framework to link microbial biogeography with its control over biogeochemical cycling, which should be explicitly incorporated into biogeochemical models predicting ecosystem functioning responses to future environmental changes.

### Conflict of interest statement

The authors declare that the research was conducted in the absence of any commercial or financial relationships that could be construed as a potential conflict of interest.
