# Supplementary material for: The large-scale distribution of ammonia oxidizers in paddy soils is driven by soil pH, geographic distance, and climatic factors
Source: Front Microbiol. 2015 Sep 4;6:938. doi: 10.3389/fmicb.2015.00938 (PMC4559657; doi:10.3389/fmicb.2015.00938)

Supplementary materials

Table S1 Locations, physicochemical properties and potential nitrification rates of the 33 paddy soil samples from the 11 sites

Sample ID	*Latitude*	*Longitude*	*MAT (℃)*	*MAP (mm)*	*pH*	*H_2O%*	*OM (g kg^{-1})*	*TN (g kg^{-1})*	*C/N ratio*	*NH_4^+-N (mg kg^{-1})*	*NO_3^--N (mg kg^{-1})*	*Sand (%)*	*Clay (%)*	*Sulfate (mg kg^{-1})*	*Chloride (mg kg^{-1})*	*PNR (mg NO_2^--N kg^{-1} dry soil h^{-1})*
LN1	41.52	123.40	8.1	717	6.84	43.1	15.5	0.55	16.4	34.2	3.67	38.5	8.69	49.7	13.8	0.15
LN2	41.52	123.40	8.1	717	6.69	38.1	13.4	0.66	11.8	38.9	0.96	33.0	9.91	131	40.6	0.33
LN3	41.52	123.40	8.1	717	6.66	37.1	16.6	0.66	14.5	15.7	1.75	41.0	1.99	56.0	10.3	0.16
PJ1	41.20	121.92	10.5	650	8.48	34.5	24.1	0.78	17.9	12.4	0.27	4.59	37.6	171	138	1.95
PJ2	41.20	121.92	10.5	650	8.77	37.9	12.8	0.60	12.3	11.7	0.38	6.86	44.0	256	163	0.39
PJ3	41.20	121.92	10.5	650	8.74	33.7	9.6	0.41	13.5	10.4	0.04	10.1	35.1	197	169	0.96
BH1	34.00	119.78	14.0	1000	8.32	40.6	30.2	1.96	8.9	9.93	2.41	19.1	27.0	193	108	12.2
BH2	34.00	119.78	14.0	1000	8.18	47.4	20.4	1.31	9.0	31.9	1.62	7.60	46.9	241	78.1	13.0
BH3	34.00	119.78	14.0	1000	8.23	42.2	12.6	0.77	9.6	27.6	1.54	12.6	13.7	355	58.8	1.64
CS1	31.50	120.60	15.4	1054	7.44	50.4	40.1	1.99	11.7	15.9	0.92	42.2	12.1	147	25.8	5.70
CS2	31.50	120.60	15.4	1054	6.85	60.4	41.1	2.01	11.9	17.6	1.12	51.6	7.77	263	30.8	10.5
CS3	31.50	120.60	15.4	1054	6.64	70.0	42.0	2.06	11.8	14.8	1.08	47.2	9.67	261	37.2	8.57
JX1	30.83	120.71	15.5	1100	6.51	71.5	24.4	1.19	11.9	38.6	1.93	15.5	26.4	145	42.0	1.56
JX2	30.83	120.71	15.5	1100	6.47	69.8	24.6	1.59	9.0	18.5	1.12	10.9	21.2	132	37.1	1.34
JX3	30.83	120.71	15.5	1100	6.56	82.9	27.4	1.76	9.1	84.6	1.07	26.0	13.9	294	52.7	2.01
XT1	30.45	113.01	16.6	1212	8.20	47.9	19.9	1.05	10.9	13.6	1.93	30.2	16.6	42.8	15.8	6.99
XT2	30.45	113.01	16.6	1212	8.21	42.9	19.3	1.26	8.9	6.93	3.82	29.8	13.0	58.7	12.3	2.98
XT3	30.45	113.01	16.6	1212	8.15	39.3	17.2	1.17	8.5	13.1	3.48	23.2	17.6	57.7	18.8	3.87
JZ1	30.42	112.18	16.3	1200	6.52	45.2	21.2	1.00	12.3	14.2	3.06	28.3	17.4	230	16.5	0.12
JZ2	30.42	112.18	16.3	1200	6.66	50.5	21.6	1.26	10.0	13.8	3.48	7.75	12.6	83.2	37.7	0.09
JZ3	30.42	112.18	16.3	1200	6.90	53.8	27.1	1.60	9.8	14.5	3.40	14.8	10.7	126	59.3	0.18
SY1	29.95	120.92	16.4	1400	5.52	64.8	32.5	1.72	10.9	34.8	5.26	11.1	13.4	58.6	14.2	0.06
SY2	29.95	120.92	16.4	1400	5.59	62.4	30.6	2.02	8.8	44.7	4.15	20.5	9.54	46.5	13.1	0.20
SY3	29.95	120.92	16.4	1400	5.06	84.5	37.8	2.72	8.1	53.9	3.99	31.7	10.0	119	15.1	0.20
ML1	28.76	113.05	17.0	1300	5.04	40.1	20.1	1.24	9.4	15.0	2.48	38.3	9.15	77.0	24.1	0.01
ML2	28.76	113.05	17.0	1300	5.07	36.6	26.1	1.66	9.1	40.9	2.69	30.7	14.7	43.2	5.27	0.01
ML3	28.76	113.05	17.0	1300	5.08	47.2	25.3	1.60	9.2	16.6	2.60	33.2	13.5	95.7	10.9	0.01
HY1	27.18	112.89	18.0	1500	5.69	55.9	25.7	1.43	10.4	16.0	0.99	33.4	11.2	134	6.85	0.01
HY2	27.18	112.89	18.0	1500	5.39	69.3	22.2	1.17	11.1	25.2	1.64	24.0	15.4	26.2	5.15	0.01
HY3	27.18	112.89	18.0	1500	5.28	86.2	28.8	1.33	12.6	24.2	3.19	25.8	18.5	67.8	10.2	0.02
XM1	24.61	117.96	21.0	1200	6.29	49.0	29.4	1.43	11.9	81.5	1.04	59.2	9.00	46.9	12.8	0.58
XM2	24.61	117.96	21.0	1200	6.76	38.4	17.9	0.84	12.4	42.6	0.62	42.6	8.88	32.3	23.6	1.26
XM3	24.61	117.96	21.0	1200	7.11	35.5	28.8	1.38	12.1	45.3	0.68	69.1	5.70	23.7	10.2	1.53

Abbreviations indicate names of the sampling sites: LN, Liaoning; PJ, Panjin; BH, Binhai; CS, Changshu; JX, Jiaxing; XT, Xiantao; JZ, Jingzhou; SY, Shangyu; ML, Miluo; HY, Hengyang; XM, Xiamen.

Table S2 Pairwise geographic distance (km) matrix

	LN	PJ	BH	CS	JX	XT	JZ	SY	ML	HY
LN										
PJ	128									
BH	894	822								
CS	1141	1084	288							
JX	1212	1157	363	75						
XT	1542	1437	748	732	737					
JZ	1591	1483	817	811	817	80				
SY	1305	1253	463	175	100	762	841			
ML	1699	1599	864	787	773	188	203	773		
HY	1859	1763	1004	887	861	364	367	842	176	
XM	1945	1880	1058	808	742	812	861	661	671	582

Table S3 The relationships between latitude, climatic factors, and soil physicochemical properties in paddy soil samples (*n* = 33) tested using Spearman's correlations

	Latitude	MAT	MAP	pH	H_2O%	OM	TN	C/N	NH_4^+-N	NO_3^--N	Sand%	Clay%	Sulfate
Latitude													
MAT	**-0.973**												
MAP	**-0.856**	**0.831**											
pH	**0.518**	**-0.531**	**-0.712**										
H_2O%	**-0.375**	0.215	**0.461**	**-0.510**									
OM	**-0.456**	0.296	**0.375**	**-0.392**	**0.567**								
TN	**-0.520**	0.295	**0.427**	**-0.441**	**0.597**	**0.889**							
C/N	**0.512**	-0.226	**-0.426**	0.236	-0.279	-0.189	**-0.571**						
NH_4^+-N	-0.305	0.292	0.257	-0.322	**0.369**	0.180	0.241	-0.116					
NO_3^--N	-0.214	0.197	**0.543**	**-0.390**	0.256	0.145	0.326	**0.367**	-0.047				
Sand%	-0.315	**0.345**	0.177	-0.289	-0.062	**0.368**	0.207	0.118	0.313	-0.183			
Clay%	0.249	-0.207	-0.149	**0.552**	-0.152	-0.309	-0.282	0.113	-0.249	-0.290	**-0.679**		
Sulfate	**0.568**	-0.335	**-0.535**	**0.412**	0.094	0.041	0.026	-0.030	-0.020	**-0.387**	-0.297	**0.413**	
Chloride	**0.642**	**-0.667**	**-0.714**	**0.676**	-0.308	-0.320	-0.339	-0.341	-0.227	**-0.460**	**-0.557**	**0.789**	**0.555**

Bold values indicate a significant difference at $P < 0.05$.

Table S4 The relationships between latitude, climatic factors, soil physicochemical properties, PNR, the abundance of AOA and AOB, and the relative abundance of AOA lineages in paddy soil samples tested using Spearman's correlations

	PNR	AOA abundance	AOB abundance	AOA community		
				%*Nitropumilus*	%*Nitrososphaera*	%*Nitrosotalea*
Latitude	**0.468**	0.035	0.338	0.288	**0.575**	**-0.770**
MAT	**-0.366**	0.044	-0.254	-0.266	**-0.494**	**0.727**
MAP	**-0.518**	-0.024	**-0.418**	-0.318	**-0.667**	**0.814**
pH	**0.687**	0.087	**0.595**	0.308	**0.810**	**-0.867**
H_2O%	-0.040	-0.074	-0.244	-0.015	-0.213	0.198
OM	0.087	0.221	0.016	-0.151	-0.143	0.123
TN	0.120	**0.349**	0.089	-0.118	-0.156	0.155
C/N	0.112	-0.284	0.086	0.110	0.110	-0.219
NH_4^+-N	-0.186	-0.116	-0.251	0.225	**-0.444**	0.254
NO_3^--N	-0.318	0.182	-0.143	0.004	**-0.445**	**0.427**
Sand%	-0.044	**0.424**	0.265	0.322	-0.334	0.077
Clay%	0.193	-0.235	-0.222	**-0.474**	**0.417**	-0.111
Sulfate	**0.409**	0.004	0.110	-0.034	**0.465**	**-0.508**
Chloride	**0.537**	-0.051	0.265	0.032	**0.731**	**-0.672**
PNR	—	**0.406**	**0.724**	0.338	**0.828**	**-762**

Bold values indicate a significant difference at $P < 0.05$.

Table S5 The relationships between latitude, climatic factors, soil physicochemical properties and the relative abundance of AOB clusters in paddy soil samples tested using Spearman's correlations

	Nitrosospira									*Nitrosomonas*	
	C 1	C 2	C 3a.1	C 3a.2	C 3b	C 4	C 9	C 10	C 11	C 6	C 7
Latitude	0.282	**0.458**	0.290	0.087	0.332	**0.389**	0.172	-0.270	**-0.447**	-0.177	-0.017
MAT	**-0.400**	**-0.527**	-0.184	-0.096	-0.278	-0.311	**-0.350**	0.148	**0.362**	0.197	0.093
MAP	-0.168	**-0.354**	-0.096	-0.313	-0.243	-0.203	-0.261	0.288	**0.353**	0.021	0.009
pH	-0.062	0.179	0.294	**0.397**	**0.362**	**0.423**	0.112	0.027	**-0.471**	0.169	**0.429**
H_2O%	0.305	-0.188	0.216	-0.134	0.155	0.032	0.005	-0.033	0.066	-0.086	-0.299
OM	-0.051	**-0.355**	0.031	-0.035	0.163	-0.039	-0.012	-0.054	0.124	0.026	-0.227
TN	-0.124	**-0.437**	0.034	-0.078	0.103	0.050	-0.130	0.002	0.131	-0.065	0.176
C/N	0.271	**0.436**	-0.087	**0.423**	0.061	-0.151	**0.404**	-0.226	-0.036	0.184	-0.131
NH_4^+-N	-0.074	-0.058	-0.294	0.039	**-0.380**	-0.305	-0.006	-0.131	**0.519**	-0.016	-0.164
NO_3^--N	0.048	-0.038	0.091	**-0.386**	-0.234	0.145	**0.405**	0.049	0.210	-0.187	-0.266
Sand%	0.059	-0.028	-0.130	-0.167	0.069	0.118	0.266	0.184	0.089	0.040	**-0.429**
Clay%	-0.154	-0.273	0.243	0.175	0.260	-0.180	**-0.384**	-0.075	-0.218	0.142	**0.533**
Sulfate	0.106	0.067	0.223	0.234	**0.360**	0.136	0.008	-0.314	**-0.368**	-0.152	**0.425**
Chloride	-0.027	0.041	0.155	0.345	0.167	0.149	-0.030	-0.166	**-0.388**	0.027	0.092
PNR	-0.107	-0.120	**0.508**	**0.407**	**0.476**	**0.441**	0.041	0.031	**-0.461**	0.104	0.184

Bold values indicate a significant difference at $P < 0.05$.

Table S6 The relationships between latitude, climatic factors, soil physicochemical properties and OTU richness and Shannon diversity of AOA and AOB in paddy soil samples tested using Spearman's correlation analysis

	AOA OTU richness	AOA Shannon index	AOB OTU richness	AOB Shannon index
Latitude	**-0.525**	**-0.537**	**0.535**	**0.548**
MAT	**0.392**	**0.246**	**-0.520**	**-0.531**
MAP	0.240	-0.082	-0.184	-0.168
pH	-0.008	0.195	**0.536**	**0.465**
H_2O%	0.097	-0.032	-0.231	-0.171
OM	0.017	0.015	-0.199	-0.180
TN	-0.193	-0.075	-0.212	-0.103
C/N	-0.207	-0.341	0.221	0.187
NH_4^+-N	0.151	-0.004	-0.198	-0.202
NO_3^--N	0.200	0.286	-0.125	-0.123
Sand%	0.009	0.251	-0.160	-0.096
Clay%	-0.072	-0.179	0.283	0.118
Sulfate	**-0.423**	**-0.464**	0.089	0.378
Chloride	-0.156	-0.111	0.139	0.349
PNR	-0.271	0.363	0.255	0.260

Bold values indicate a significant difference at $P < 0.05$.

Figure S1 Neighbour-joining phylogenetic tree showing the phylogenetic affiliations of the representative sequences of AOA *amoA* gene retrieved from the 33 paddy soils collected from China. The scale bar represents 5% nucleic acid sequence divergence, and bootstrap values (> 50%) are indicated at branch points.

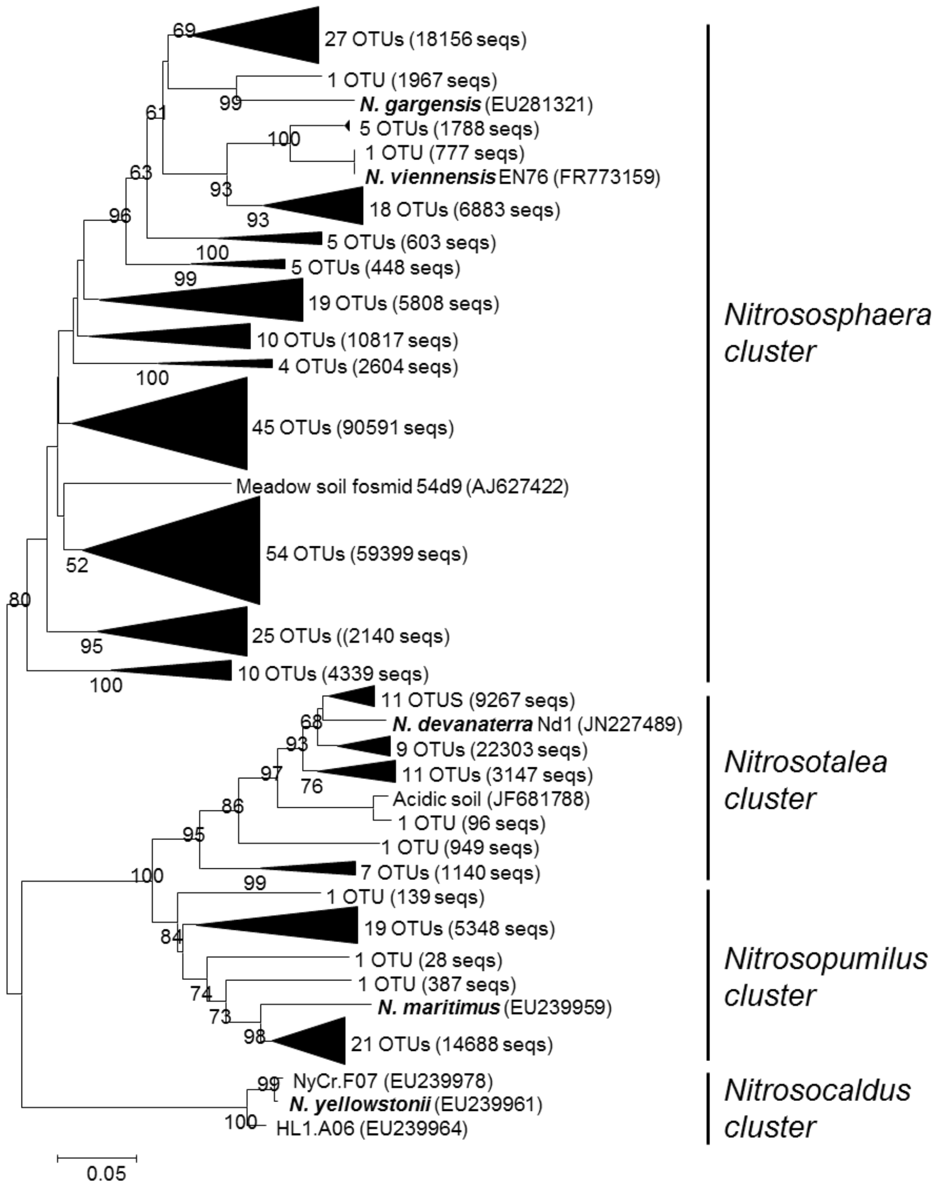

Figure S2 Neighbour-joining phylogenetic tree showing the phylogenetic affiliations of the representative sequences of AOB *amoA* gene retrieved from the 33 paddy soils collected from China. The scale bar represents 5% nucleic acid sequence divergence, and bootstrap values (> 50%) are indicated at branch points.

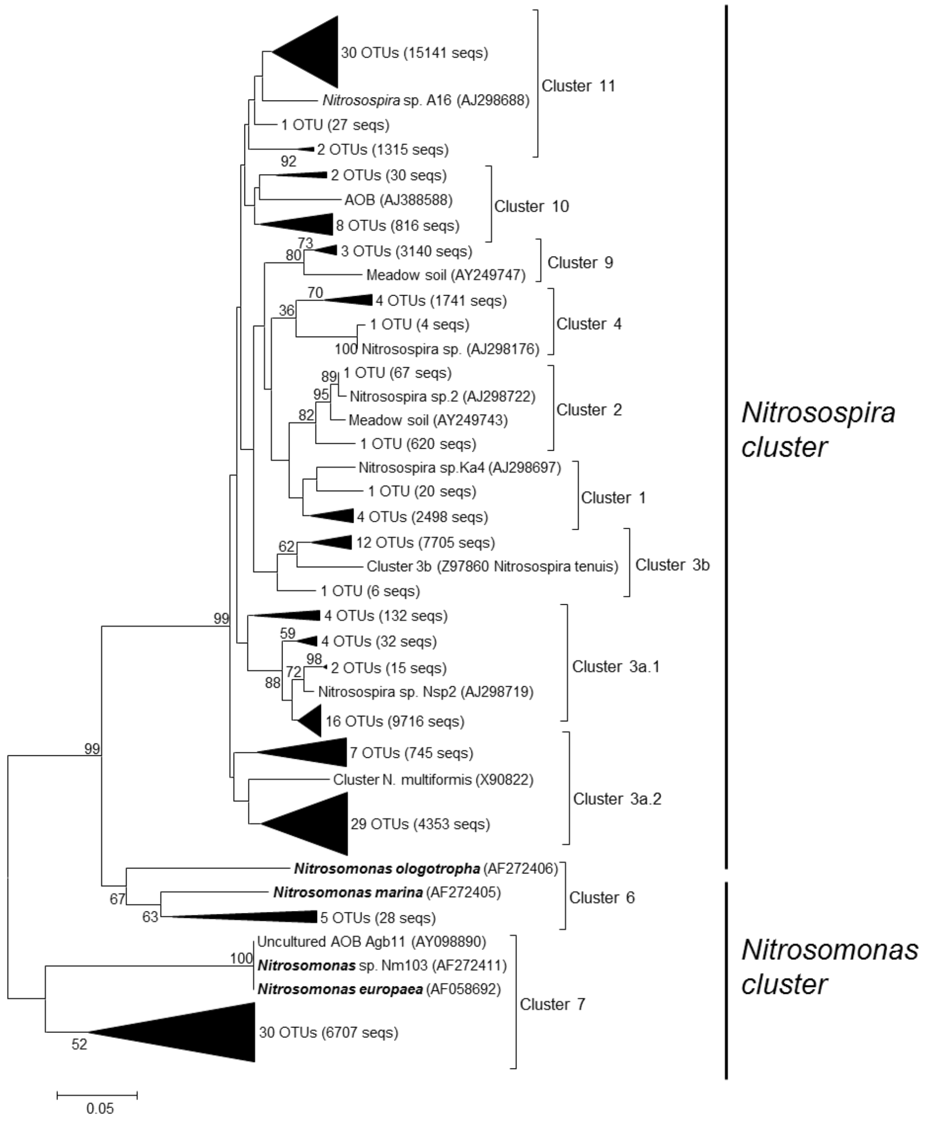

Supplement: Supplementary file 1 [file Presentation1.PDF]
